# Correction to ‘POT-3 preferentially binds the terminal DNA-repeat on the telomeric G-overhang’

**DOI:** 10.1093/nar/gkae242

**Published:** 2024-04-03

*Nucleic Acids Research*, Volume 51, Issue 2, 25 January 2023, Pages 610–618, https://doi.org/10.1093/nar/gkac1203

The authors wish to report an error in supplementary Figure 1 of their article. The middle panel says that the syb2415 mutation is a 500bp deletion that introduces a premature stop codon. Syb2415 is indeed a 500bp deletion spanning the region indicated in the figure, but it does not introduce a premature stop codon.

The supplementary file of the published article has been updated.

This change does not affect the results, discussion and conclusions presented in the article.

