# Supplementary material for: POT-3 preferentially binds the terminal DNA-repeat on the telomeric G-overhang
Source: Nucleic Acids Res. 2022 Dec 30;51(2):610–8. doi: 10.1093/nar/gkac1203 (PMC9881156; doi:10.1093/nar/gkac1203)
Supplement: gkac1203_Supplemental_File [file gkac1203_supplemental_file.pdf]

Supplementary Figure 1

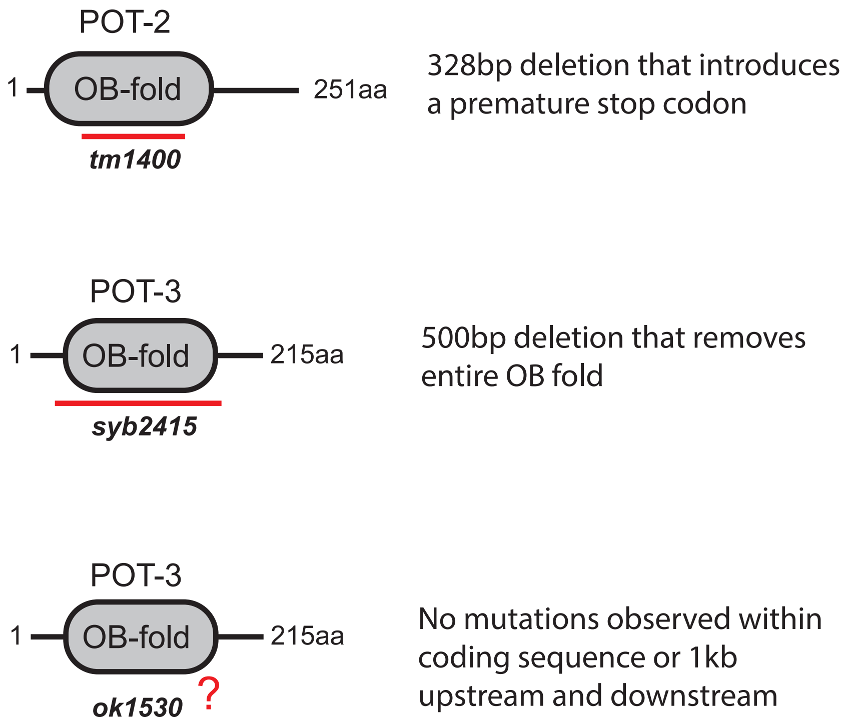

Supplementary Figure 2

A

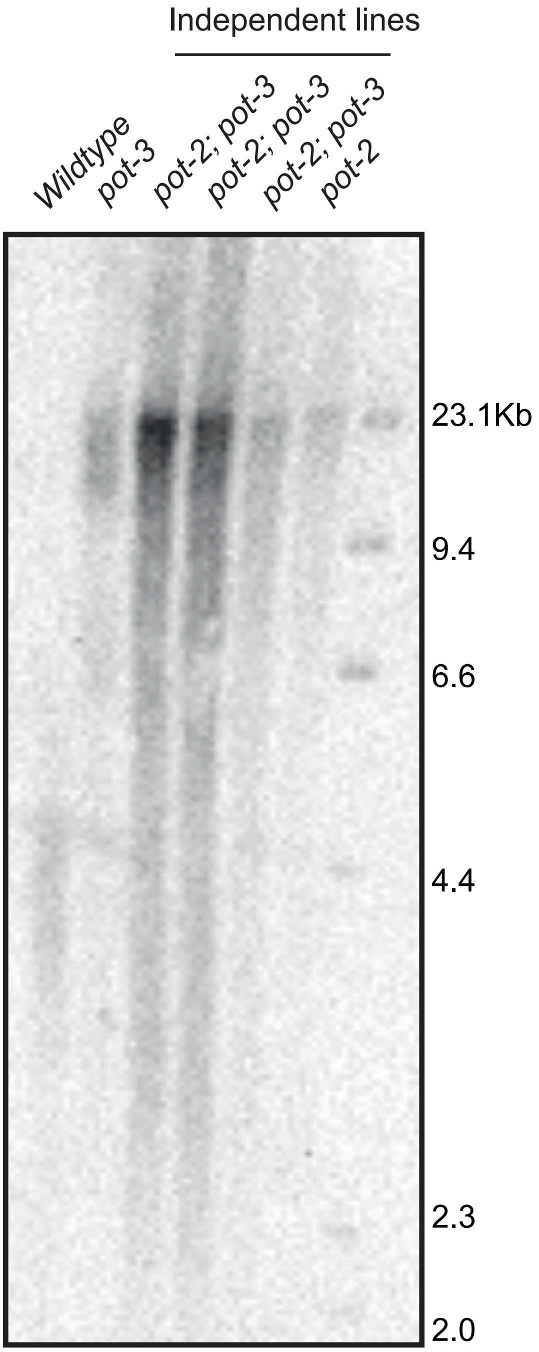

Supplementary Figure 3

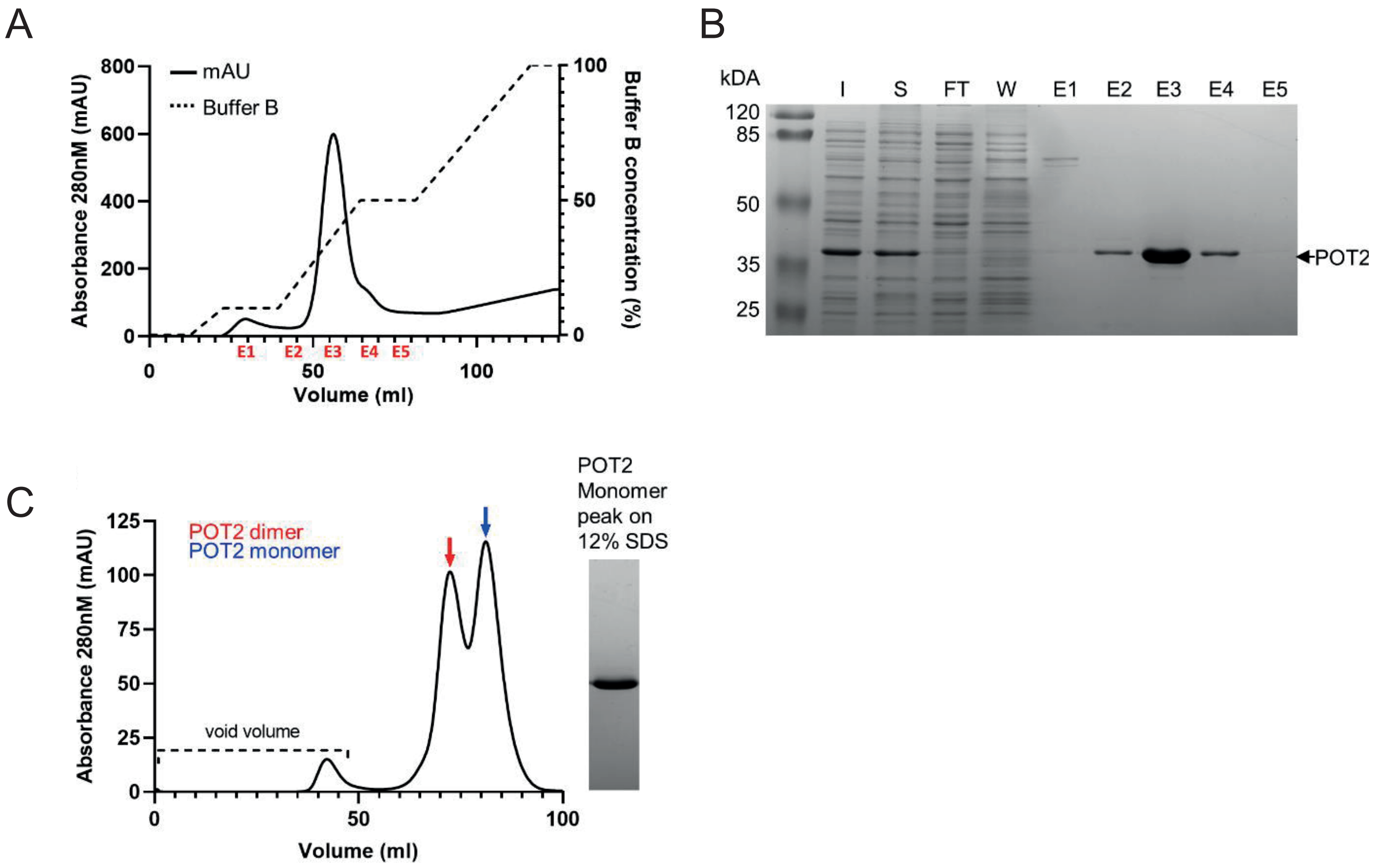

Supplementary Figure 4

A

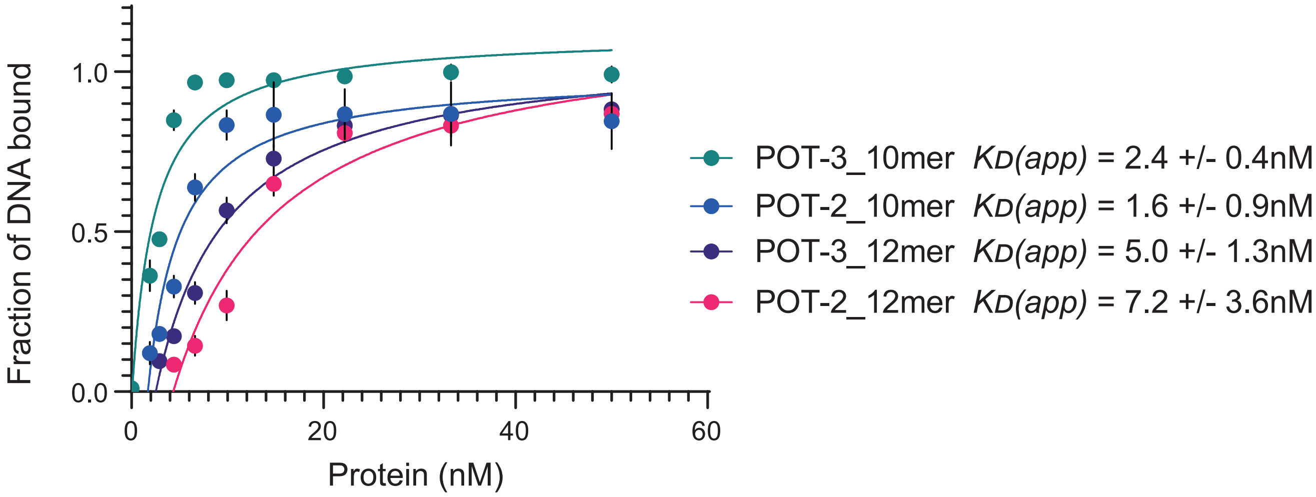

Supplementary figure 5

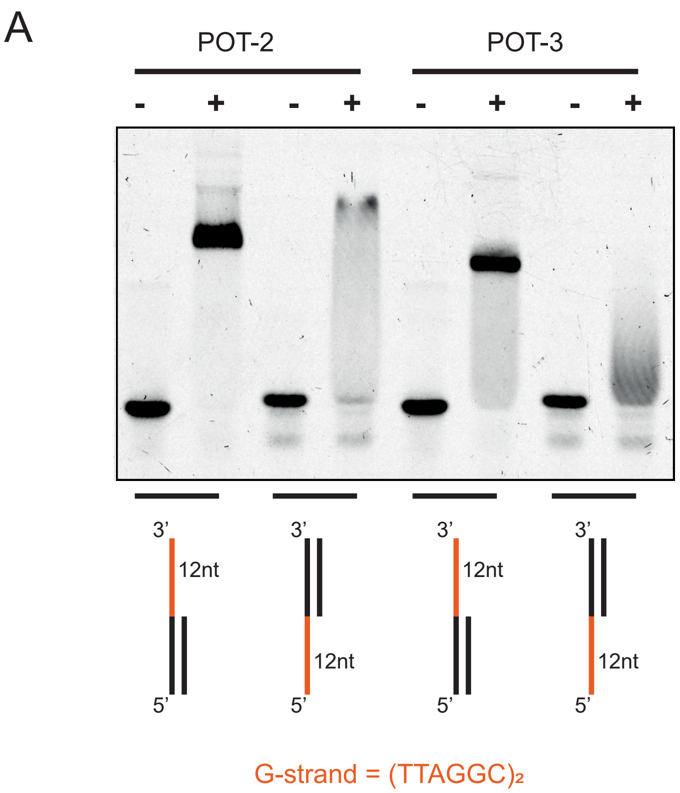

Supplementary figure 6

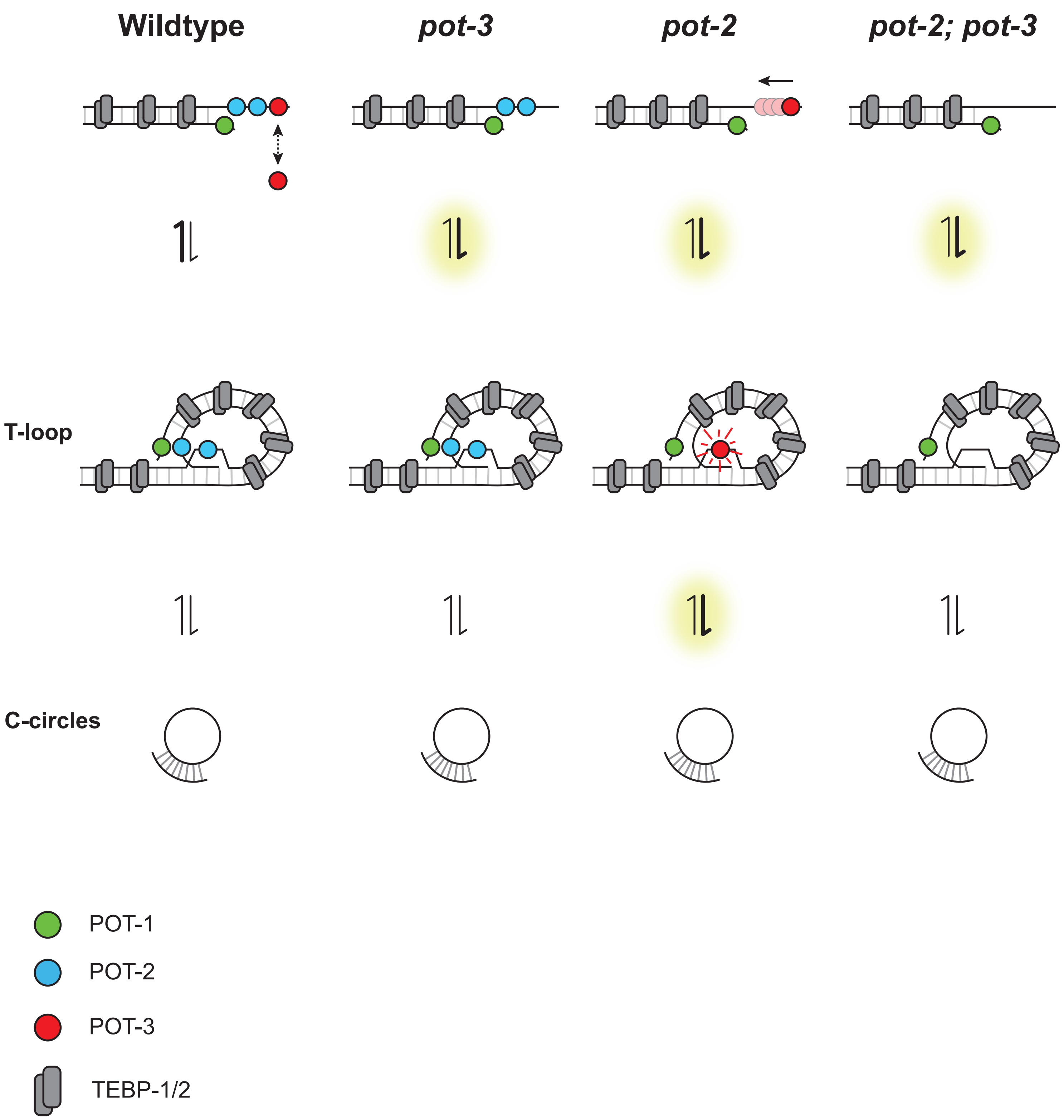

Supplementary Figure S1. Illustration of alleles used and the regions of the protein affected by mutation. *pot-2(tm1400)* and *pot-3(syb2415)* are null alleles as they contain large deletions than remove the majority of the OB fold. Sequencing *pot-3(ok1530)* failed to detect any mutations within the *pot-3* coding sequence nor 1kb upstream or downstream of this region. This allele appears to be wildtype for POT-3.

Supplementary Figure S2. *pot-2; pot-3* double mutants show reproducible behaviour across independent lines. Multiple *pot-2; pot-3* double mutants were isolated from crossing *pot-2* and *pot-3* single mutants. All double mutants have reproducibly long telomeres that more heterogeneous and slightly shorter than *pot-2*.

Supplementary Figure S3. Purification of 6xHis-tagged POT-2 expressed from *E. coli*. **A.** Trace of Nickel affinity chromatography on Akta purifier. **B.** Coomassie stained SDS-PAGE gel of different fractions collected during the purification: I - insoluble, S - soluble, FT - flowthrough, W - wash E1-5 - elution fractions. **C.** Affinity purified POT-2 was subsequently run over a HiLoad 16/60 Superdex 200 size exclusion column. The majority of POT-2 migrates as a monomer although it can form disulphide-mediated protein dimers.

Supplementary Figure 4. POT-2 and POT-3 show weaker affinity for DNA at room temperature and both bind 10mer DNA tighter than 12mer DNA. 0.2nM Cy5-labelled DNA was incubated with increasing amounts of either POT-2 or POT3 in binding buffer (50mM NaCl, 20mM Tris- HCl pH8.0, 50µg/mL BSA, 1mM $MgCl_2$, 5mM DTT, 0.001% Tween-20), for 1 hour at room temperature. After this, Ficoll 400 was added to a final concentration of 4% and the reaction cooled on ice briefly before running on 7% native polyacrylamide gels in cold 0.5x TBE buffer. Each data point shows the average and standard deviation of three independent experiments. Apparent binding affinities (K_D) were calculated using Prism 9.4.1 by fitting the data to a modified 'specific binding total' equation where the non-specific component was set to 0. Data points showing less than 10% binding where excluded due to inaccuracies in measuring the low amount of signal.

Supplementary Figure 5. POT-3 requires its recognition sequence to be close to a 3' end of DNA. POT2 or POT3 (500nM) was incubated with Cy5 labelled oligonucleotide substrate (50nM) containing a non-specific duplex with TTAGGCTTAGCC overhangs at either the 3' or 5' end.

Supplementary Figure 6. Speculative model to explain the epistatic relationship between *pot-2* and *pot-3*. In wildtype worms, POT-3 binds the end of the G-overhang. It needs to be removed from the G overhang to reveal the terminal telomeric ssDNA that is able to invade upstream telomeric dsDNA and form a T-loop. This is unfavourable and therefore relatively few T-loops are formed. The inappropriate processing of T-loops via DNA repair pathways may facilitate the formation of C-circles. In *pot-3* worms, the end of the G-overhang is more accessible and this shifts the equilibrium (yellow highlight) to higher levels of T-loops and consequently higher levels of C-circles. The precise mechanism by which T-loops are processed into C-circles is unclear. However, it may require initial G-overhang invasion and processing to expose a sufficiently long C-overhang that can then form a stable T-loop via C-overhang invasion. Endonucleolytic cleavage and ligation of this structure may then produce a C-circle. In *pot-2* worms, there is not enough POT-3 to coat the G overhang and POT-3 binding is no longer restricted to 3' end. Therefore, as in *pot-3* mutants, the increased accessibility of the 3' end increases T-loop formation (yellow highlight). However, the remaining POT-3 may now bind inappropriately to the displaced G-strand within the T-loop. We speculate that this makes the T-loop more recombinogenic, shifting the equilibrium (yellow highlight) towards the higher levels of C-circles. This increase in C-circle levels in *pot-2* can be suppressed by removing POT-3, such that a *pot-2, pot-3* double mutant now has lower C-circle levels than in the single *pot-2* mutant and instead now shows similar C-circle levels to a single *pot-3* mutant.

Supplementary table 1

Molecular weight ladder calibration curve (From Figure 1B)

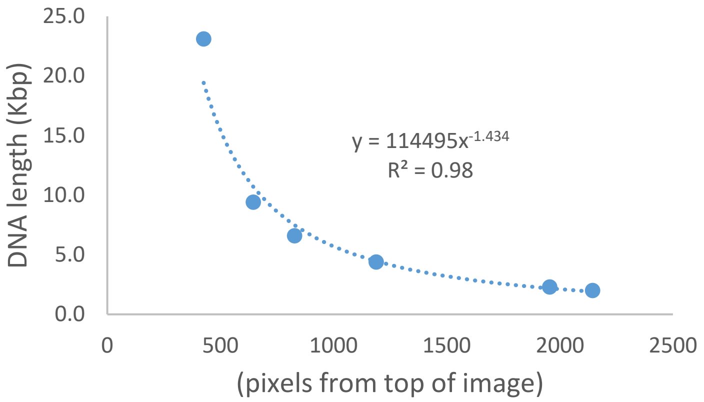

Telomere length

Strain	Mode (Kbp)	Range (Kbp)
Wildtype	3.9	2.2 - 8.6
pot-2	18.1	6.9 - 52.5
pot-3	9.8	5.5 - 23.9
pot-2; pot-3	13.7	6.3 - 21.3

Supplementary table 2

Strain list

	Strain Name	Genotype	Reference
Wildtype	N2	*Bristol strain N2*	
pot-2	HFW2 (CeOB1)	*pot-2(tm1400)*	1
pot-3	HFW103	*pot-3(syb2415)*	This paper
pot-2; pot-3	HFW109	*pot-2(tm1400); pot-3(syb2415)*	This paper
trt-1 (ALT survivor)	HFW36 (c1-25)	*trt-1(ok410) unc-29(e193)*	2

1. Raices, M., et al., *C. elegans* telomeres contain G-strand and C-strand overhangs that are bound by distinct proteins. Cell, 2008. 132(5): p. 745-57.

2. Cheng, C et al., *Caenorhabditis elegans* POT-2 telomere protein represses a mode of alternative lengthening of telomeres with normal telomere lengths. Proc. Natl. Acad. Sci. 109, 7805–7810 (2012).
